# Supplementary material for: MetaRibo-Seq measures translation in microbiomes
Source: Nat Commun. 2020 Jun 29;11:3268. doi: 10.1038/s41467-020-17081-z (PMC7324362; doi:10.1038/s41467-020-17081-z)
Supplement: Supplementary file 10 — Supplementary Data 7 [file 41467_2020_17081_MOESM10_ESM.zip › File2/Confidence_High_Taxonomy/378064_out.krona.html]

Javascript must be enabled to view this page.

members
magnitude
magnitudeUnassigned
count
unassigned
taxon
rank

378064\_out

5

superkingdom
5
2

phylum
5
976

200643
5
class

171549
5
order

family
5
815

genus
816
5


SRS013158\_contig\_number\_3167SRS015694\_contig\_number\_22497SRS023971\_contig\_number\_contig-100\_188.42055SRS062654\_contig\_number\_17865SRS098644\_contig\_number\_27574
species
821
5
